# Incidence of possible serious bacterial infection in young infants in the three high-burden countries of the Democratic Republic of the Congo, Kenya, and Nigeria: A secondary analysis of a large, multi-country, multi-centre clinical trial

**DOI:** 10.7189/jogh.14.04009

**Published:** 2024-02-02

**Authors:** Adrien Lokangaka, Manimaran Ramani, Melissa Bauserman, Jackie Patterson, Cyril Engmann, Antoinette Tshefu, Simons Cousens, Shamim Ahmad Qazi, Adejumoke Idowu Ayede, Ebunoluwa A Adejuyigbe, Fabian Esamai, Robinson D Wammanda, Yasir Bin Nisar, Yves Coppieters

**Affiliations:** 1 Kinshasa School of Public Health, Université de Kinshasa, Kinshasa, Democratic Republic of the Congo; 2 School of Public Health, Université Libre de Bruxelles, Brussels, Belgium; 3 University of Alabama at Birmingham, Birmingham, Alabama, USA; 4 University of South Alabama, Birmingham, Alabama, USA; 5 University of North Carolina at Chapel Hill, Chapel Hill, North Carolina, USA; 6 University of Washington, Seattle, Washington, USA; 7 PATH Organization, Seattle, Washington, USA; 8 Department of Infectious Disease Epidemiology, London School of Hygiene and Tropical Medicine (LSHTM), London, United Kingdom; 9 Newborn and Child Health Consultant, Geneva, Switzerland; 10 Department of Paediatrics, College of Medicine, University of Ibadan, and University College Hospital, Ibadan, Nigeria; 11 Department of Paediatrics and Child Health, Obafemi Awolowo University, Ile-Ife, Nigeria; 12 Department of Child Health and Paediatrics, School of Medicine, Moi University, Eldoret, Kenya; 13 Department of Paediatrics, Ahmadu Bello University Teaching Hospital, Ahmadu Bello University, Zaria, Nigeria; 14 Department of Maternal, Newborn, Child and Adolescent Health and Ageing, World Health Organization, Geneva, Switzerland

## Abstract

**Background:**

Neonatal infections are a major public health concern worldwide, particularly in low- and middle-income countries, where most of the infection-related deaths in under-five children occur. Sub-Saharan Africa has the highest mortality rates, but there is a lack of data on the incidence of sepsis from this region, hindering efforts to improve child survival. We aimed to determine the incidence of possible serious bacterial infection (PSBI) in young infants in three high-burden countries in Africa.

**Methods:**

This is a secondary analysis of data from the African Neonatal Sepsis (AFRINEST) trial, conducted in the Democratic Republic of the Congo (DRC), Kenya, and Nigeria between 15 March 2012 and 15 July 2013. We recorded baseline characteristics, the incidence of PSBI (as defined by the World Health Organization), and the incidence of local infections among infants from 0–59 days after birth. We report descriptive statistics.

**Results:**

The incidence of PSBI among 0–59-day-old infants across all three countries was 11.2% (95% confidence interval (CI) = 11.0–11.4). The DRC had the highest incidence of PSBI (19.0%; 95% CI = 18.2–19.8). Likewise, PSBI rates were higher in low birth weight infants (24.5%; 95% CI = 23.1–26.0) and infants born to mothers aged <20 years (14.1%; 95% CI = 13.4–14.8). The incidence of PSBI was higher among infants delivered at home (11.7%; 95% CI = 11.4–12.0).

**Conclusions:**

The high burden of PSBI among young infants in DRC, Kenya, and Nigeria demonstrates the importance of addressing PSBI in improving child survival in sub-Saharan Africa to reach the Sustainable Development Goals (SDGs). These data can support government authorities, policymakers, programme implementers, non-governmental organisations, and international partners in reducing preventable under-five deaths.

**Registration:**

Australian New Zealand Clinical Trials Registry: ACTRN12610000286044.

The risk of dying among under-five children is highest in the first month of life [1]. Neonatal infections are responsible for 7.5% (0.4 million) of under-five-year-old childhood deaths worldwide, most of which occur in low- and middle-income countries (LMICs) [1]. The Sustainable Development Goals (SDG) for 2030 define a target under-five mortality rate of 25 or fewer deaths per 1000 live births [2]. This rate remains high in sub-Saharan Africa at 74 deaths per 1000 live births, making it one of the regions with the highest burden of child mortality globally [1,3]. Though progress has been made worldwide toward achieving SDG child survival goals, analyses conducted in 2019 prior to the coronavirus disease 2019 (COVID-19) pandemic suggest that sub-Saharan African countries must double their current progress rate to achieve the SDG target by 2030 [3]. With the pandemic’s negative effects on maternal, newborn, and child health programmes, these projections are likely now gross underestimations [4,5]. Given the proportion of under-five children who die due to infection-related causes, targeted efforts to reduce infection-related mortality in sub-Saharan Africa are critical to accelerating progress. An important barrier to this progress is a detailed characterisation of possible serious bacterial infection (PSBI).

The incidence of PSBI among young infants in LMICs varies notably between regions [6] and is significantly less than values reported from high-income countries (HICs). Studies from sub-Saharan Africa, South Asia, and Latin America found a pooled estimate of PSBI incidence of around 7.6% [7]. Hospital-acquired neonatal infections in LMICs have been reported to range from 6.5 to 38 per 1000 live births, which is 3–20 times higher than the rates reported in HICs [8]. Home-based studies from Bangladesh and India found that approximately 10% of all newborns had an illness with at least one sign of PSBI during routine follow-up visits [9,10]. Determining an accurate incidence of PSBI is crucial for developing and evaluating interventions to improve neonatal mortality.

The signs and symptoms of neonatal bacterial infection are non-specific, making the diagnosis of sepsis in neonates a challenge. In 2019, the Integrated Management of Childhood Illness (IMCI) algorithm to identify young infants with PSBI for hospital referral was modified for low-resource settings [11,12]. The sensitivity and specificity of this algorithm are high, at 87% and 74%, respectively, when it is used by first-level health workers [13]. We sought to use data from the African Neonatal Sepsis (AFRINEST) trial to report the incidence of PSBI in communities in the Democratic Republic of the Congo (DRC), Kenya, and Nigeria using the IMCI algorithm, and relate participant characteristics to the incidence of sepsis.

## METHODS

In the AFRINEST Trial (parent study), we enrolled pregnant mothers, neonates, and young infants up to 59 days of age in the three African countries: the DRC, Kenya, and Nigeria. The full details of the AFRINEST trial have been published elsewhere [14,15].

### Study setting

The DRC, Kenya, and Nigeria represented Eastern, Central, and Western African countries, respectively. In the DRC, we conducted the study in rural areas of four health zones in the North and South Ubangi provinces, with a study area population of approximately 300 000. Each health zone serves between 100 000 to 250 000 inhabitants and consists of 8–24 health areas. In turn, each health area serves a population of 5000 to 10 000 inhabitants through one health centre, with the possibility of referral to a general hospital. In Kenya, we conducted the study in 34 clusters from Busia, Bungoma, and Kakamega counties (estimated total population: 350 000). Each cluster operates under the care of a Chief and serves a population of about 10 000. In Nigeria, we conducted the study in Ibadan, Ile-Ife, and Zaria. In Ibadan, we conducted the study in Ido and Lagelu, peri-urban local government areas of Oyo State in southwestern Nigeria (estimated total population: 200 000). Most people lived in rural areas. In Ile-Ife, we conducted the study in the Central and Eastern local government areas of the Ile-Ife semi-urban area located in Osun State, in southwestern Nigeria (estimated total population: 350 000). In Zaria, we conducted the study in the Zaria local government areas, located in the north of Kaduna State (estimated total population: 150 000).

### Study period

All three countries have rainy and dry seasons. The rainy season is between mid-April to mid-November for the DRC sites, March to September for Kenya sites, April to October for Ibadan and Ile–Ife in Nigeria, and May to November for Zaria, Nigeria. To capture the seasonal variations in the incidence of infections in the participating sites, we enrolled participants in this study for over one year (15 March 2012 and 15 July 2013).

### Surveillance

#### Pregnancy surveillance

Pregnancy surveillance was conducted using the same procedures and survey instruments across all sites. The surveillance activities aimed to identify newborns on the first day of life. Community health workers (CHWs) in DRC and Kenya and community health extension workers (CHEWs) in Nigeria identified pregnant mothers through two home visits. The first visit was carried out at around five months of pregnancy, and the second at four weeks before the anticipated delivery date or as soon as possible if the pregnancy was identified later than seven months. The CHWs/CHEWs used pre-designed surveillance forms during each home visit, which were the same in all sites and adapted from the relevant World Health Organization (WHO) tools [16].

#### Newborn surveillance

The CHWs/CHEWs visited pregnant mothers within 24 hours after birth to assess the infants for PSBI and conducted additional visits to evaluate the infant on days 3, 7, 14, 21, 28, 35, 42, 49, and 60 after birth. Low birth weight infants had one additional visit on day 2. If the baby was unavailable on a specified day, a repeat visit was made on the following day. Using pre-designed surveillance forms adapted from WHO tools, CHWs/CHEWs assessed the infant for signs of PSBI at each home visit and counselled families on the signs of infection, such as not feeding well, convulsions, fast breathing (60 breaths or more per minute), severe chest indrawing, temperature ≥37.5°C or <35.5°C, movement only with stimulation, yellow soles or draining pus from the umbilicus, eye, or skin [12].

### Identification of sick newborns and young infants

Young infants were considered to have a PSBI if they presented with one or more of the following according to IMCI guidelines for neonates and young infants: axillary temperature >37.5°C or <35.5°C, stopped feeding well, movement only when stimulated, severe chest indrawing, or fast breathing. Upon recognising one or more signs or symptoms of infection, the infant was referred to a health centre for further assessment by a trained health worker, who then reassessed the infant. If they confirmed that the infant had clinical signs of infection, they would refer the infant to a hospital for treatment. Those who refused referral to the health centre or could not access one were further assessed by a study-trained health worker using the IMCI tool [12]. Parents of these infants were also asked for consent to participate in a randomised trial of treatment for severe infection [14,15].

### Training

Trainers from the Department of Maternal Child Health and Adolescent Health (MCA) at the WHO conducted master trainer workshops for study investigators and managers from all sites using the ‘WHO/UNICEF Home Care for Newborns,’ ‘WHO/UNICEF Young Infant IMCI’ (for study nurses), and ‘Study specific procedures’ courses [15,16]. All CHWs/CHEWs and nurses employed in the study were trained at their respective sites by these master trainers. All supervisors and site coordinators were trained similarly by the master trainers on-site before the commencement of the study. More details have been published elsewhere [14,15]. We repeated the same training for all staff, including the nurses, supervisors, and coordinators, every six months throughout the study period. The staff were also trained in the identification of signs of critical illness.

### Quality assurance

#### Standardisation

Extensive standardisation exercises were conducted for each cadre of study health workers. For the CHWs/CHEWs standardisation sessions, we identified 5-10 eligible young infants in a community or a nearby health facility and asked each CHW/CHEW to assess them while being observed by the trained facilitator. To establish the trainees’ competencies, evaluators or trainers assessed the infants before assigning them to the trainees, whom they then supervised at the time of assessment. The assessments included weighing the child; assessing danger signs (ability to feed, convulsions, counting respiratory rate, and identifying the presence of severe chest indrawing); taking a temperature and identifying whether it is normal, high, or low; assessing for movement; and checking for yellow soles.

Trainees who needed further standardisation were given additional targeted, individualised refresher training. Standardisation exercises were conducted at the start of the study and subsequently every six months for all health workers to ensure that all of them similarly assessed clinical signs.

#### Supervision

The programme managers and investigators conducted the supervision at each site on a weekly and monthly basis. The nurses supervised the CHWs/CHEWs at the cluster level, and the cluster supervisors were the overall supervisors for the cluster study personnel (CHWs/CHEWs and treatment/enrolment nurses). The programme managers supervised the cluster coordinators and had overall oversight of all the clusters in conducting the study. The principal investigators and co-investigators made random visits to check quality and compliance with the protocol and standard operating procedures.

### Data collection

During home visits, the CHWs/CHEWs collected data on all births and deaths within the first two months after birth and assessed infants for signs of PSBI. The data was collected on standard paper forms which were used during the pilot study and extensively tested in the field and revised as necessary. The case report forms did not record the name or other information which would allow the identification of the infant or family. Study supervisors checked all forms for completeness before entering them into a computer using the same standard operating procedures at all study sites.

### Data analysis

Each site was responsible for its own day-to-day data management activities and had a data management team comprising a data manager and a team of data entry operators. At each site, the data entry clerks entered the data from the paper forms into the data management system designed for the study, consisting of a front-end and a back-end. The front-end, designed in C++, was the user interface used by the data manager and data entry clerks, while the database itself was stored in the back-end in SQL. It was based on a double-entry system, where discrepancies were checked in order to minimise data entry errors. One of the data manager’s tasks was to perform range and consistency checks integrated with the data management system. After resolving all range and consistency errors in consultation with the study coordinators and data collection staff, the data manager checked for inconsistencies in information on different forms using other built-in checks and resolved them in consultation with the field-based study coordinators and, if necessary, with the study’s principal investigators.

The cleaned data was sent every month to the central data coordination centre at the London School of Hygiene and Tropical Medicine, London, UK, where additional data quality checks were carried out and feedback was provided to the study sites every month.

### Ethical considerations

Communities in all sites were sensitised to the study before launch through meetings with community leaders and community groups. We obtained written informed consent for home visits for pregnancy and birth surveillance and the infants’ follow-up visit. The local institutional review boards at all study sites, the WHO Ethical Review Committeee, and the London School of Hygiene and Tropical Medicine Institutional Review Board approved the study.

## RESULTS

**Figure 1:**
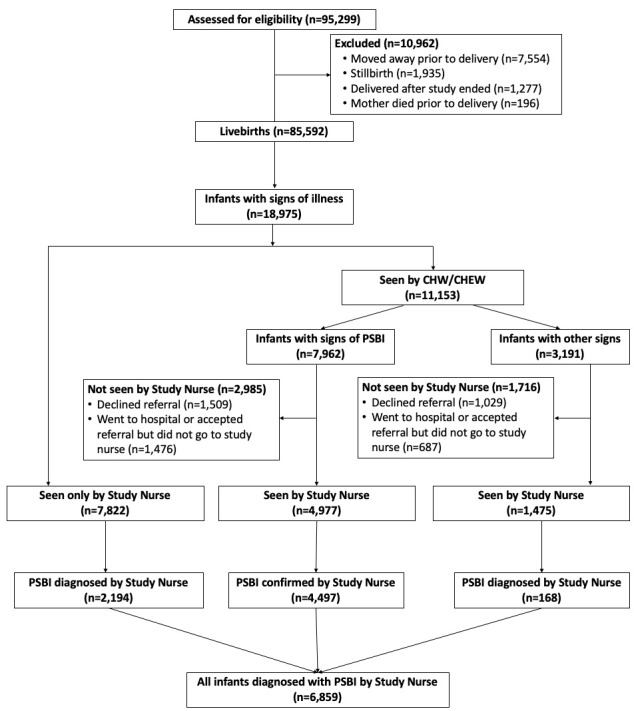
Flow diagram.

We identified 95 299 pregnancies during the 16-month study period (**Figure 1**). Among these pregnant women, 7554 mothers moved away before delivery, 196 died before CHW/CHEW follow-up, 1277 were still pregnant when the study ended, and 1935 were stillbirths during the study period. CHWs/CHEWs identified 85 592 live births. Among these, 7822 (9.1%) sought direct care from a study nurse, of which 2194 infants (2.6%) were found to have PSBI. Among 11 153 (13.0%) infants identified with danger signs by CHWs/CHEWs, 7962 (9.3%) were classified by the CHW/CHEW as having PSBI and 3191 (3.7%) as having other signs such as local infection, jaundice, or low birth weight. Overall, 2985 (3.5%) infants referred by CHWs/CHEWs for PSBI confirmation were not seen by the study nurse. Of 4977 infants identified as having any sign of PSBI by CHW/CHEW, 4479 (5.2%) also saw a study nurse who confirmed the diagnosis. Of 3191 (3.7%) infants identified by the CHWs/CHEWs as having other signs, PSBI was confirmed by the study nurse in 168 infants. The study nurse did not see 1716 (2.0%) infants referred by CHWs/CHEWs for other signs (**Figure 1**).

Most enrolled infants (93.7%) weighed 2.5 kg or more; the DRC had 12.1% low birth weight infants compared to a range of 3.9–7.7% in the other sites. Nearly half of all births (48.6%; n = 41 534) occurred at a health facility; the proportion of home deliveries was 65.8% in Kenya and 77.4% in Zaria (Nigeria) compared to a range of 19.1–46.3% at the other sites. Fifty-one per cent of all infants were male (n = 43 614). The proportion of mothers aged less than 20 years was 18.4% in Kenya and 17.4% in the DRC compared to a range of 4.0–11.7% in the other sites (**Table 1**).

**Table 1:** Infant baseline characteristics, presented as n (%)

Characteristics	DRC (n = 9087)	Kenya (n = 22 101)	Ibadan (n = 18 871)	Ile-Ife (n = 24 120)	Zaria (n = 11 413)	All sites (n = 85 592)	*P*-Value
**Birth weight in kgs**							0.03
<2.5	1032 (12.1)	437 (3.9)	806 (5.2)	499 (4.5)	879 (7.7)	3653 (6.3)	
≥2.5	7517 (87.9)	10 649 (96.1)	14 732 (94.8)	10 609 (95.5)	10 528 (92.3)	54 035 (93.7)	
**Place of birth**							0.01
Facility	7199 (80.9)	7562 (34.2)	10 125 (53.7)	14 074 (58.3)	2583 (22.6)	41 543 (48.6)	
Home	1697 (19.1)	14 539 (65.8)	8746 (46.3)	10 046 (41.7)	8830 (77.4)	43 858 (51.4)	
**Sex**							0.09
Male	4698 (51.7)	11 063 (50.1)	9705 (51.4)	12 306 (51.0)	5842 (51.2)	43 614 (51.0)	
Female	4389 (48.3)	11 038 (49.9)	9166 (48.6)	11 814 (49.0)	5571 (48.8)	41 978 (49.0)	
**Maternal age in y**							0.04
<20	1576 (17.4)	4070 (18.4)	751 (4.0)	1279 (5.3)	1332 (11.7)	9008 (10.5)	
≥20	7481 (82.6)	18 027 (81.6)	18 115 (96.0)	22 841 (94.7)	10 081 (88.3)	76 545 (89.5)	

DRC – Democratic Republic of Congo, y – years

Ten scheduled home visits were completed in 49.0% of infants (n = 42 079), and 38.3% (n = 32 750) received between five and nine home visits in the first two months after birth by CHWs/CHEWs (**Table 2**). Completion of home visits was fairly similar across sites.

**Table 2:** Home visits made by CHWs or CHEWs for young infants in the first two months after birth, presented as n (%)

Number of home visits	DRC (n = 9087)	Kenya (n = 22 101)	Ibadan (n = 18 871)	Ile-Ife (n = 24 120)	Zaria (n = 11 413)	All sites (n = 85 592)	*P*-value
Ten	5501 (60.5)	11 654 (52.7)	10 902 (57.8)	8459 (35.1)	5563 (48.7)	42 079 (49.0)	0.04
Nine	783 (8.6)	3691 (16.7)	763 (4.0)	2399 (10.0)	1755 (15.4)	9391 (11.0)	0.01
Eight	560 (6.2)	2256 (10.2)	1219 (6.5)	3069 (12.7)	1281 (11.2)	8385 (9.8)	0.03
Seven	495 (5.5)	1389 (6.3)	1479 (7.8)	2218 (9.2)	810 (7.1)	6391 (7.5)	0.04
Six	464 (5.1)	889 (4.0)	1069 (5.7)	1622 (6.7)	496 (4.4)	4540 (5.3)	0.10
Five	508 (5.6)	688 (3.1)	875 (4.6)	1583 (6.6)	389 (3.4)	4043 (4.7)	0.03
Four	368 (4.1)	598 (2.7)	950 (5.0)	1712 (7.1)	384 (3.4)	4012 (4.7)	0.04
Three	215 (2.4)	463 (2.1)	790 (4.2)	1707 (7.1)	325 (2.9)	3500 (4.1)	0.04
Two	142 (1.6)	372 (1.7)	716 (3.8)	1277 (5.3)	261 (2.3)	2768 (3.2)	0.06
One	51 (0.6)	101 (0.5)	108 (0.6)	74 (0.3)	149 (1.3)	483 (0.6)	0.23

DRC – Democratic Republic of Congo

The overall incidence of PSBI was 11.2% (95% confidence interval (CI) = 11.0–11.4) among 0–59-day-old infants, 4.6% (95% CI = 4.4–4.7) among infants aged 0–6 days, and 9.2% (95% CI = 9.0–9.4) among those 0–27 days of age (**Table 3**). The DRC had an incidence of PSBI of 19.0% (95% CI = 18.2–19.8) among 0–59-day-old infants, 9.0% (95% CI = 8.4–9.6) among 0–6 days-old infants, and 16.1% (95% CI = 15.3–16.8) among 0–27-day-old infants. The incidence of PSBI among low birth weight infants aged 0-59 days old at all sites combined was 24.5% (95% CI = 23.1–26.0) compared to 11.2% (95% CI = 11.0–11.5) for infants ≥2.5 kg. For low birth weight infants 0–6 days old, the incidence of PSBI was 16.6% (95% CI = 15.4–17.9) compared to 5.1% (95% CI = 4.9–5.2) for infants ≥2.5 kg. For low birth weight infants 0–27 days old, the incidence of PSBI was 22.0% (95% CI =  20.7–23.4) compared to 9.5% (95% CI = 9.3–9.8) for infants ≥2.5 kg (**Table 3**). The incidence of PSBI for home deliveries was 11.7% (95% CI = 11.4–12.0) for infants 0–59 days of age, 4.8% (95% CI = 4.6–5.0) for those 0–6 days of age, and 9.7% (95% CI = 9.4–10.0) for those 0–27 days of age. These annual rates are compared to facility birth PSBI incidence of 10.6% (95% CI = 10.3-10.9) for infants aged 0–59 days, 4.4% (95% CI = 4.2–4.6) for infants 0-6 days old, and 8.6% (95% CI = 8.4–8.9) for infants 0–27 days old. In the DRC, however, the incidence of PSBI in home births for infants 0–59 days was 18% compared to 19.2% in facility births. In all sites combined, the incidence of PSBI among infants aged 0–59 days born to mothers <20 years old was 14.1% (95% CI = 13.4–14.8) compared to 10.8% (95% CI = 10.6–11.0) for infants born to mothers ≥20 years of age.

**Table 3:** Incidence of PSBI by infant characteristics and postnatal days, presented as n (%; 95% CI)

Characteristic	Postnatal days	DRC (n = 9087)	Kenya (n = 22 101)	Ibadan (n = 18 871)	Ile-Ife (n = 24 120)	Zaria (n = 11 413)	All sites (n = 85 592)	*P*-value
Overall	0–6	818 (9.0; 8.4–9.6)	989 (4.5; 4.2–4.8)	721 (3.8; 3.6–4.1)	676 (2.8; 2.6–3.0)	720 (6.3; 5.9–6.8)	3924 (4.6; 4.4–4.7)	0.03
	0–27	1460 (16.1; 15.3–16.8)	2499 (11.3; 10.9–11.7)	1322 (7.0; 6.6–7.4)	1215 (5.0; 4.8–5.3)	1382 (12.1; 11.5–12.7)	7878 (9.2; 9.0–9.4)	
	0–59	1728 (19.0; 18.2–19.8)	3193 (14.4; 14.0–14.9)	1663 (8.8; 8.4–9.2)	1382 (5.7; 5.4–6.0)	1590 (13.9; 13.3–14.6)	9556 (11.2; 11.0–11.4)	
Birth weight in kg								
*<2.5*	0–6	187 (18.1; 15.8–20.6)	94 (21.5; 17.7–25.7)	91 (11.3; 9.2–13.7)	79 (15.8; 12.7–19.3)	157 (17.9; 15.4–20.6)	608 (16.6; 15.4–17.9)	0.02
	0–27	245 (23.7; 21.2–26.5)	119 (27.2; 23.1–31.7)	135 (16.7; 14.2–19.5)	94 (18.8; 15.5–22.5)	212 (24.1; 21.3–27.1)	805 (22.0; 20.7–23.4)	
	0–59	271 (26.3; 23.6–29.1)	131 (30.0; 25.7–34.5)	158 (19.6; 16.9–22.5)	103 (20.6; 17.2–24.5)	233 (26.5; 23.6–29.6)	896 (24.5; 23.1–26.0)	
*≥2.5*	0–6	603 (8.0; 7.4–8.7)	518 (4.9; 4.5–5.3)	603 (4.1; 3.8–4.4)	448 (4.2; 3.8–4.6)	563 (5.3; 4.9–5.8)	2735 (5.1; 4.9–5.2)	0.03
	0–27	1142 (15.2; 14.4–16.0)	1109 (10.4; 9.8–11.0)	1055 (7.2; 6.8–7.6)	675 (6.4; 5.9–6.8)	1170 (11.1; 10.5–11.7)	5151 (9.5; 9.3–9.8)	
	0–59	1353 (18.0; 17.1–18.9)	1315 (12.3; 11.7–13.0)	1281 (8.7; 8.2–9.2)	759 (7.2; 6.7–7.7)	1357 (12.9; 12.3–13.5)	6065 (11.2; 11.0–11.5)	
Place of birth								
*Facility*	0–6	676 (9.4; 8.7–10.1)	298 (3.9; 3.5–4.4)	336 (3.3; 3.0–3.7)	342 (2.4; 2.2–2.7)	169 (6.5; 5.6–7.6)	1821 (4.4; 4.2–4.6)	0.04
	0–27	1181 (16.4; 15.6–17.3)	795 (10.5; 9.8–11.2)	669 (6.6; 6.1–7.1)	658 (4.7; 4.3–5.0)	287 (11.1; 9.9–12.4)	3590 (8.6; 8.4–8.9)	
	0–59	1380 (19.2; 18.3–20.1)	1061 (14.0; 13.3–14.8)	844 (8.3; 7.8–8.9)	763 (5.4; 5.1–5.8)	342 (13.2; 12.0–14.6)	4390 (10.6; 10.3–10.9)	
*Home*	0–6	125 (7.4; 6.2–8.7)	691 (4.8; 4.4–5.1)	385 (4.4; 4.0–4.9)	334 (3.3; 3.0–3.7)	551 (6.2; 5.7–6.8)	2086 (4.8; 4.6–5.0)	0.01
	0–27	244 (14.4; 12.7–16.1)	1704 (11.7; 11.2–12.3)	653 (7.5; 6.9–8.0)	557 (5.5; 5.1–6.0)	1095 (12.4; 11.7–13.1)	4253 (9.7; 9.4–10.0)	
	0–59	306 (18.0; 16.2–19.9)	2132 (14.7; 14.1–15.2)	819 (9.4; 8.8–10.0)	619 (6.2; 5.7–6.6)	1248 (14.1; 13.4–14.9)	5124 (11.7; 11.4–12.0)	
Sex								
*Male*	0–6	467 (9.9; 9.1–10.8)	517 (4.7; 4.3–5.1)	396 (4.1; 3.7–4.5)	347 (2.8; 2.5–3.1)	410 (7.0; 6.4–7.7)	2137 (4.9; 4.7–5.1)	0.03
	0–27	810 (17.2; 16.2–18.4)	1328 (12.0; 11.4–12.6)	748 (7.7; 7.2–8.3)	621 (5.0; 4.7–5.4)	797 (13.6; 12.8–14.5)	4304 (9.9; 9.6–10.2)	
	0–59	938 (20.0; 18.8–21.1)	1710 (15.5; 14.8–16.1)	928 (9.6; 9.0–10.2)	720 (5.9; 5.4–6.3)	910 (15.6; 14.7–16.5)	5206 (11.9; 11.6–12.2)	
*Female*	0–6	351 (8.0; 7.2–8.8)	472 (4.3; 3.9–4.7)	325 (3.5; 3.2–3.9)	329 (2.8; 2.5–3.1)	310 (5.6; 5.0–6.2)	1787 (4.3; 4.1–4.5)	0.02
	0–27	650 (14.8; 13.8–15.9)	1171 (10.6; 10.0–11.2)	574 (6.3; 5.8–6.8)	594 (5.0; 4.6–5.4)	585 (10.5; 9.7–11.3)	3574 (8.5; 8.2–8.8)	
	0–59	790 (18.0; 16.9–19.2)	1483 (13.4; 12.8–14.1)	735 (8.0; 7.5–8.6)	662 (5.6; 5.2–6.0)	680 (12.2; 11.4–13.1)	4350 (10.4; 10.1–10.7)	
Maternal age in y								
*<20*	0–6	159 (10.1; 8.6–11.7)	215 (5.3; 4.6–6.0)	31 (4.1; 2.8–5.8)	35 (2.7; 1.9–3.8)	96 (7.2; 5.9–8.7)	536 (6.0; 5.5–6.5)	0.03
	0–27	288 (18.3; 16.4–20.3)	480 (11.8; 10.8–12.8)	50 (6.7; 5.0–8.7)	67 (5.2; 4.1–6.6)	175 (13.1; 11.4–15.1)	1060 (11.8; 11.1–12.5)	
	0–59	337 (21.4; 19.4–23.5)	602 (14.8; 13.7–15.9)	64 (8.5; 6.6–10.8)	73 (5.7; 4.5–7.1)	194 (14.6; 12.7–16.6)	1270 (14.1; 13.4–14.8)	
*≥20*	0–6	658 (8.8; 8.2–9.5)	774 (4.3; 4.0–4.6)	690 (3.8; 3.5–4.1)	641 (2.8; 2.6–3.0)	624 (6.2; 5.7–6.7)	3387 (4.4; 4.3–4.6)	0.04
	0–27	1166 (15.6;14.8–16.4)	2019 (11.2; 10.7–11.7)	1272 (7.0; 6.7–7.4)	1148 (5.0; 4.7–5.3)	1207 (12.0; 11.3–12.6)	6812 (8.9; 8.7–9.1)	
	0–59	1383 (18.5; 17.6–19.4)	2590 (14.4; 13.9–14.9)	1599 (8.8; 8.4–9.2)	1309 (5.7; 5.4–6.0)	1396 (13.8; 13.2–14.5)	8277 (10.8; 10.6–11.0)	

DRC – Democratic Republic of Congo, y – years

Regarding local infections, skin infections predominated at all sites among 0–27 days old and 0–59 days old, 4.4%, and 5.1%, respectively. In the early neonatal period, the incidence of eye infection at all sites was 1.1% (95% CI = 1.0–1.1) compared to a 0.9% rate of skin infections and a 0.7% rate of umbilical infections in the same period (**Table 4**). Umbilical infections in Nigeria had an annual incidence of <1% for all three postnatal subgroups.

**Table 4:** Incidence of local infection by postnatal days, presented as n (%; 95% CI)

Type of Infection	Postnatal days	DRC (n = 9087)	Kenya (n = 22 101)	Ibadan (n = 18 871)	Ile-Ife (n = 24 120)	Zaria (n = 11 413)	All sites (n = 85 592)	*P*-value
Skin	0–6	66 (0.7; 0.6–0.9)	498 (2.3; 2.1–2.5)	30 (0.2; 0.1–0.2)	52 (0.2; 0.2–0.3)	140 (1.2; 1.0–1.4)	786 (0.9; 0.9–1.0)	0.03
	0–27	228 (2.5; 2.2–2.9)	2457 (11.1; 10.7–11.5)	128 (0.7; 0.6–0.8)	181 (0.8; 0.6–0.9)	801 (7.0; 6.6–7.5)	3795 (4.4; 4.3–4.6)	
	0–59	267 (2.9; 2.6–3.3)	2863 (13.0; 12.5–13.4)	145 (0.8; 0.6–0.9)	200 (0.8; 0.7–1.0)	870 (7.6; 7.1–8.1)	4345 (5.1; 4.9–5.2)	
Umbilicus	0–6	59 (0.6; 0.5–0.8)	476 (2.2; 2.0–2.4)	3 (0.0; 0.0–0.0)	43 (0.2; 0.1–0.2)	41 (0.4; 0.3–0.5)	622 (0.7; 0.7–0.8)	0.02
	0–27	167 (1.8; 1.6–2.1)	1269 (5.7; 5.4–6.1)	9 (0.0; 0.0–0.1)	61 (0.3; 0.2–0.3)	77 (0.7; 0.5–0.8)	1583 (1.8; 1.8–1.9)	
	0–59	201 (2.2; 1.9–2.5)	1435 (6.5; 6.2–6.8)	11 (0.1; 0.0–0.1)	61 (0.3; 0.2–0.3)	89 (0.8; 0.6–1.0)	1797 (2.1; 2.0–2.2)	
Eye	0–6	274 (3.0; 2.7–3.4)	289 (1.3; 1.2–1.5)	44 (0.2; 0.2–0.3)	73 (0.3; 0.2–0.4)	223 (2.0; 1.7–2.2)	903 (1.1; 1.0–1.1)	0.04
	0–27	641 (7.1; 6.5–7.6)	694 (3.1; 2.9–3.4)	56 (0.3; 0.2–0.4)	104 (0.4; 0.4–0.5)	379 (3.3; 3.0–3.7)	1874 (2.2; 2.1–2.3)	
	0–59	783 (8.6; 8.0–9.2)	813 (3.7; 3.4–3.9)	58 (0.3; 0.2–0.4)	106 (0.4; 0.4–0.5)	410 (3.6; 3.3–4.0)	2170 (2.5; 2.4–2.6)	

DR – Democratic Republic of Congo

## DISCUSSION

This is a large multi-centre study representing Eastern, Central, and Western Africa that reports a community-based annual incidence of PSBI among young infants in these three sub-Saharan African countries, with a combined annual incidence of 11.2%. We found an incidence of PSBI of 19% in the DRC, 14.4% in Kenya, and 10.2% in Nigeria. In other parts of Africa, the incidence of neonatal infection has been reported to vary from 6.5 to 23 per 1000 live births, much lower than we observed in our study [17,18]. Our findings fall within the range of the cohort study conducted in six LMICs participating in the National Institute of Child Health and Human Development Global Network's Maternal and Newborn Health Registry, between 1 January 2010 and 31 December 2013, where the incidence of PSBI varied from 3% in Zambia to 36% in Pakistan [19]. Our study also showed an incidence of PSBI of 24.5% among low birth weight infants, while other studies reported incidences of 25% and 55.84% [20,21].

We observed that babies born to mothers under 20 years of age had a significantly higher incidence of PSBI compared to 6% for those born to older mothers (14.1% vs 6.0%; *P* = 0.03). Previous studies have also reported a correlation between maternal age and poor neonatal outcomes [21–23]. Concerning the place of delivery, we found that infants delivered at home had a significantly higher incidence of PSBI than those delivered at the facility (11.7% vs 10.6%; *P* = 0.01), in line with other studies [24–26]. In our study, however, the incidence of PSBI was slightly higher among infants born in health facilities in the DRC. Poor sanitation and unhygienic conditions in health facilities are common, and many health centres and hospitals in rural DRC lack water and sanitation [27]. Furthermore, we found a significantly higher incidence of PSBI among male infants than females (11.9% vs 10.4%; *P* = 0.03), which is consistent with the published literature. Cross-sectional and observational, analytical, and retrospective studies conducted at Tishreen University Hospital in Syria [21] and in Peru [28] identified male sex as one of the risk factors for developing early-onset sepsis [20].

The three most common cases of neonatal deaths are preterm birth, intrapartum-related events (i.e. birth asphyxia) and infections such as sepsis/PSBI, meningitis, and pneumonia [1,29]. The SDG agenda to end preventable child deaths will not be met without a significant reduction in neonatal deaths; thus, it is critical to improve the quality of maternal intrapartum care and immediate newborn care to address neonatal infections. As far as PSBI/sepsis management is concerned, a series of trials were carried out to increase treatment coverage in young infants up to two months of age when a referral is not possible [15,30–32]. A large proportion of families do not accept referral advice for hospitalisation due to various reasons [33–36]. To increase the coverage of treatment for PSBI when a referral to a hospital is not feasible, the WHO developed a guideline to manage such young infants. However, calculating the correct coverage and impact of the WHO guideline requires good incidence data, which is lacking in most places. Our study contributes to filling this gap in sub-Saharan Africa.

To increase coverage of PSBI treatment using the WHO guideline [37], a few sites evaluated the feasibility of delivering simplified antibiotic regimens to young infants with PSBI where a referral was not feasible. This innovative approach included careful assessments and measurements of the logistical requirements for program uptake and expansion and evaluated the feasibility, facilitators, and barriers to implementation and scale-up of the WHO guideline. This intervention took place in Bangladesh, the DRC, Ethiopia, India, Malawi, Nigeria, and Pakistan [27,34–36,38–46]. The WHO PSBI guideline was successfully implemented within the programme setting in all sites and achieved high treatment coverage [47]. This has the potential to address high neonatal mortality and morbidity in LMICs, even though it will require government commitment to establish an enabling environment in the health system by strengthening it with well-trained, motivated, and paid health care providers. For a successful scale-up of the intervention, necessary medicines and supplies should be provided, and technical support for implementation and strengthening liaison with communities should be put in place.

Our study has several strengths. We report a population-level incidence of PSBI ascertained in a scalable, but rigorous manner in three countries representing Eastern, Central and Western Africa. This study shows the importance of the contribution of CHWs/CHEWs in identifying PSBI in local communities. It was conducted within the context of the existing health structure, the protocol benefited from highly-quality supervision, and trained and standardised study nurses confirmed the eligibility criteria. Nonetheless, our study also had some limitations. No microbiology was performed, and the incidence of malaria, a seasonally mediated condition, was not determined. Our assessment of PSBI was limited to young infants up to two months of age.

## CONCLUSIONS

The high incidence of PSBI among young infants in the DRC, Nigeria, and Kenya observed in our study should compel government authorities, non-governmental organisations, and international partners to collaborate to end preventable deaths due to PSBI to achieve one of the SDGs targeting infant and child mortality.
